# Neonatal Lipopolysaccharide Exposure Delays Puberty and Alters Hypothalamic *Kiss1* and *Kiss1r* mRNA Expression in the Female Rat

**DOI:** 10.1111/j.1365-2826.2009.01885.x

**Published:** 2009-08

**Authors:** A M I Knox, X F Li, J S Kinsey-Jones, E S Wilkinson, X Q Wu, YS Cheng, S R Milligan, S L Lightman, K T O'Byrne

**Affiliations:** * Division of Reproduction & Endocrinology, King's College LondonLondon, UK; † Henry Wellcome Laboratory for Integrative Neuroscience & Endocrinology, University of BristolBristol, UK

**Keywords:** neonatal, LPS, puberty, kisspeptin, Kiss1, GPR54, rat

## Abstract

Immunological challenge experienced in early life can have long-term programming effects on the hypothalamic-pituitary-adrenal axis that permanently influence the stress response. Similarly, neonatal exposure to immunological stress enhances stress-induced suppression of the hypothalamic-pituitary gonadal (HPG) axis in adulthood, but may also affect earlier development, including the timing of puberty. To investigate the timing of the critical window for this programming of the HPG axis, neonatal female rats were injected with lipopolysaccharide (LPS; 50 μg/kg i.p.) or saline on postnatal days 3 + 5, 7 + 9, or 14 + 16 and monitored for vaginal opening and first vaginal oestrus as markers of puberty. We also investigated the effects of neonatal programming on the development of the expression patterns of kisspeptin (Kiss1) and its receptor (Kiss1r) in hypothalamic sites known to contain kisspeptin-expressing neuronal populations critical to reproductive function: the medial preoptic area (mPOA) and the arcuate nucleus in neonatally-stressed animals. We determined that the critical period for a significant delay in puberty as a result of neonatal LPS exposure is before 7 days of age in the female rat, and demonstrated that *Kiss1*, but not *Kiss1r* mRNA, expression in the mPOA is down-regulated in pre-pubertal females. These data suggest that the mPOA population of kisspeptin neurones play a pivotal role in controlling the onset of puberty, and that their function can be affected by neonatal stress.

Exposure to an immune challenge in the perinatal period has been shown to have profound and long-lasting effects on the stress response throughout later life. Neonatal rats treated with lipopolysaccharide (LPS) show a marked increase in sensitivity to stress as adults compared to their saline-treated counterparts, revealing a long-term programming of the hypothalamic-pituitary-adrenal (HPA) axis responses by this early stress (1–3). The mechanism underlying this effect of LPS is not fully understood, although LPS administration mimics events occurring during infectious stress, including the induction of the febrile response, increased production of cytokines such as interleukin-1β, interleukin-6 and tumour necrosis factor-α leading to increased cyclooxygenase-2 and prostaglandin-E2 (PGE2) production (4, 5). Nevertheless, neonatal LPS exposure is associated with increased corticotrophin-releasing factor (CRF) gene expression in the paraventricular nucleus of the hypothalamus, and an increase in the pulse frequency and amplitude of corticosterone release in adulthood (1, 6). However, the critical time period for this neonatal programming is not known.

There has been relatively little research into the interaction of neonatal immune challenges with the hypothalamic-pituitary-gonadal (HPG) axis. Our own study (7) has examined the effects of LPS administration to neonates on pulsatile luteinising hormone (LH) secretion and CRF and CRF receptor expression in the medial preoptic area (mPOA), and found that an immune challenge on postnatal days 3 and 5 provokes heightened sensitivity of the gonadotrophin-releasing hormone (GnRH) pulse generator to the inhibitory influence of stress in adulthood. The HPG axis, and hence the GnRH pulse generator, is now thought to be under the central control of the kisspeptin (Kiss1) and Kiss1r (otherwise known as GPR54, the G-coupled receptor for kisspeptin) signalling system in the hypothalamus. Kisspeptin was established as a potent activator of the reproductive system via Kiss1r mediation in 2005 (8). This system was linked to the function of the HPG axis after it was noticed that mutations in the *Kiss1r* gene result in hypogonadotrophic hypogonadism (9, 10), and that Kiss1r knockout mice exhibit the same disorder (10). Kisspeptin/Kiss1r signalling is considered part of the hypothalamic circuitry that governs the hypothalamic secretion of GnRH (11) and is strongly implicated as a ‘gatekeeper’ for the initiation of puberty. Peripheral kisspeptin administration stimulates precocious puberty in rats (12–14) and sustained gonadotrophic hormone secretion in juvenile rhesus monkeys (15). Furthermore, polymorphisms in the *Kiss1* gene and activating mutations in *Kiss1r* have been associated with precocious puberty in humans (16, 17). Although the mechanisms controlling the timing of kisspeptin/Kiss1r activation at puberty remain to be established, it has recently been shown that 17β-oestradiol (E_2_) is important for driving the increase in hypothalamic kisspeptin expression prior to puberty in the mouse (18).

Kisspeptin neurones are localised in several nuclei of the hypothalamus. Two of these are crucial for the regulation of gonadotrophic hormone secretion in the rat: the mPOA, which includes the anteroventral periventricular nucleus (AVPV), and the arcuate nucleus (ARC). During the onset of puberty, *Kiss1* and *Kiss1r* expression increases in the hypothalamus in both male and female rats (13); a further study in mice suggests that there is differential expression of *Kiss1* from neurones in the ARC and AVPV during puberty: the expression shows an increase in the AVPV, but remains relatively stable in the ARC (19).

In the present study, we tested the hypotheses that a neonatal immune challenge during a certain critical time window will delay the onset of puberty, and that such an immune challenge will affect *Kiss1* and *Kiss1r* expression in the mPOA and ARC nuclei. Our results suggest that the mPOA population of kisspeptin neurones may play a pivotal role in controlling the onset of puberty, and that their function can be affected by neonatal stress.

## Materials and methods

### Animals

Pregnant Sprague-Dawley rats obtained from Charles River (Margate, UK) were housed under controlled conditions (12 : 12 h light/dark cycle, lights on 07.00 h; temperature 22 ± 2 °C) and supplied with food and water *ad lib*. Litters were reduced to a maximum of 14 pups 3 days after birth (taking the day of birth as day 0), to standardise competition for food and maternal attention and partially correct for gender imbalance. Litters were weaned at postnatal (pnd) day 21, and female offspring were housed in groups of four to six per cage. All procedures were conducted in accordance with the United Kingdom Home Office Regulations.

### Neonatal endotoxin exposure: determining a critical window for delays in puberty as a result of neonatal immune challenge

Litters were randomly divided into three groups. On pnd 3 and 5, the first group were injected with 50 μg/kg endotoxin in 0.05 ml sterile saline (LPS, serotype *Esterichia coli* 055:B5; Sigma-Aldrich, Poole, UK), a dose sufficient to effect permanent HPA axis activation (1, 6). Half the pups in each litter were designated controls, and received an i.p. injection of sterile saline (0.05 ml). The second group were injected with LPS (50 μg/kg endotoxin in 0.15 ml) or saline on pnd 7 and 9. The third group were injected with LPS (50 μg/kg endotoxin in 0.25 ml) or saline on pnd 14 and 16. Litters were weaned as described, and females were monitored daily for vaginal opening from pnd 32. Once vaginal opening occurred, a vaginal smear was taken using a steel wire loop dipped in sterile saline. Animals were monitored until first oestrus was observed, and weighed weekly.

### Neonatal endotoxin exposure: effects on *Kiss1* and *Kiss1r* expression in the mPOA and ARC

On pnd 3 and 5, a separate group of pups was injected i.p. with 50 μg/kg LPS or saline as control as described above. The animals were killed by decapitation at various time points: pnd 14, 32, on the day of vaginal opening (dVO), or pnd 77 (11 weeks of age, hereafter ‘Adult’). Animals from the latter two groups were monitored for vaginal opening and oestrus cyclicity as described above, from pnd 32; they were also weighed weekly. The adult animals were bilaterally ovariectomised at 10 weeks of age and implanted with a Silastic capsule (inner diameter 1.57 mm; outer diameter 3.18 mm; Sanitech, Havant, UK), filled to a length of 25 mm with E_2_ (Sigma-Aldrich) dissolved at a concentration of 20 μg/ml arachis oil (Sigma–Aldrich). The E_2_-containing capsules were assumed to produced circulating concentrations of E_2_ within the range observed during the dioestrous phase of the oestrous cycle (approximately 38.8 ± 1.2 pg/ml) as previously described by Maeda *et al.* (20). Surgical procedures were carried out under ketamine (100 mg/kg i.p.; Pharmacia and Upjohn Ltd, Crawley, UK) and Rompun (10 mg/kg i.p.; Bayer, Leverkusen, Germany) anaesthesia. The rationale for ovariectomy and E_2_ replacement of the adult group was to eliminate the impact of a fluctuating gonadal steroid milieu on hypothalamic *Kiss1* and *Kiss1r* mRNA expression and in addition to allow comparison with other studies carried out in our laboratory using different stress paradigms (21).

### Tissue collection and quantitative reverse trasncriptase-polymerase chain reaction (RT-PCR)

Expression of *Kiss1* and *Kiss1r* mRNA was determined by real-time quantitative RT-PCR in the mPOA and ARC from animals culled at pnd 14, pnd 32, dVO and as adults. The whole brain was carefully removed, frozen on dry ice, and stored at −80 °C. Sections were cut at 300 μm on a cryostat (Bright Ltd, Cambridgeshire, UK) for RT-PCR. Bilateral punches (1 mm in diameter) from the mPOA, which included the AVPV, were taken from bregma +0.2 to −0.4; a single midline punch (1 mm diameter) was taken from bregma −1.7 to −3.9 to include both ARC nuclei. Co-ordinates were obtained from the rat brain atlas of Paxinos and Watson (22) using the micropunch method described by Palkovits (23). The punched sections were fixed with formalin and stained with crystal violet to confirm correct punch positioning under a microscope. Total RNA was extracted from the punched mPOA and ARC tissues for each rat using TRI reagent (Sigma-Aldrich) in accordance with the manufacturer's instructions. Reverse transcription was carried out using the reverse transcriptase Superscript II (Invitrogen, Carlsbad, CA) and random primer following the manufacturer's instructions. For the qPCR, the following primers were used: Kiss1: (sense) 5′-TGGCACCTGTGGTGAACCCTGAAC-3′, (antisense) 5′-ATCAGGCGACTGCGGGTGGCACAC-3′; Kiss1r: (sense) 5′-TGTGCAAATTCGTCAACTACATCC-3′, (antisense) 5′-AGCACCGGGGCG GAAACAGCTGC-3′. 28S rRNA: (sense) 5′-TTGAAAATCCGGGGGAGAG-3′, (antisense) 5′-ACATTGTTCCAACATGCCAG-3′. The primer pairs selected for Kiss1 and Kiss1r detection were designed to amplify across at least one intron, ruling out the possibility of identical size bands resulting from genomic DNA amplification. Based on the rat Kiss1 genomic sequence (accession number: NM181692.1), the primers for Kiss1 will amplify a fragment of 192 bp corresponding to nucleotides 74–275 of the GenBank sequence. The Kiss1r sense primer corresponds to nucleotides 341–533 of the Genbank sequence (NM023992.1), with cDNA products of 194 bp. The LightCycler (Roche Biochemicals, Lewes, UK) was used for real-time quantitative analysis of *Kiss1* and *Kiss1r* mRNA expression. The sample cDNA prepared as above was used as a template for the PCR. During PCR, the amplified cDNA products were detected after each annealing phase in real time using the Faststart DNA Master SYBR Green I kit (Roche Biochemicals). Each reaction included 2 μl of sample cDNA (optimised so that sample values of the PCR product were within the standard curve), 0.5 μl each of 25 μm antisense and sense primers, 2 μl 15 mm MgCl_2_, 1 μl Faststart DNA master SYBR Green mix and 4 μl of water to give a total reaction volume of 10 μl. The Kiss1 reaction conditions were 10 min at 95 °C for one cycle, then 10 s at 95 °C, 10 s at 56 °C and 10 s at 72 °C for 32 cycles. The Kiss1r reaction conditions were 15 min at 94 °C for one cycle, then 15 s at 95 °C, 30 s at 63 °C and 16 s at 72 °C for 34 cycles. The 28S rRNA reaction conditions were 10 min at 95 °C for one cycle, then 15 s at 95 °C, 10 s at 54 °C and 5 s at 72 °C for 28 cycles. Reaction conditions for *Kiss1* and *Kiss1r* mRNA and 28S rRNA were optimised separately to give the best results for each primer and for the different quantities of target in samples. Preliminary experiments were undertaken to optimise the Mg^2+^ concentration, to confirm PCR specificity by agarose gel electrophoresis and melting curve analysis, and to prepare the PCR products used to generate standard curves in real-time PCR. Kiss1 and Kiss1r were quantified against a standard curve of samples containing known Kiss1, Kiss1r and 28S PCR product concentrations, using the LightCycler™ software. The 28S rRNA was quantified as a reference gene against a separate standard curve of samples containing known concentrations of 28S rRNA product. The melting curves for Kiss1 and Kiss1r mRNAs and 28S rRNA generated by the LightCycler™ software demonstrated that single products were amplified. PCR product for Kiss1 and Kiss1r mRNAs was sequenced and analysed using an ABI PRISM 310 (Applied Biosystems, Foster City, CA, USA).

### Statistical analysis

Comparisons between neonatal LPS and saline treatment groups on vaginal opening and first oestrus were made by subjecting data to one-way anova and Dunnett's test. A two-way anova and Newman–Keuls post-hoc analysis was used to assess the effects of LPS treatment and time on the expression of hypothalamic *Kiss1* and *Kiss1r*. P < 0.05 was considered statistically significant in all cases. Data are presented as the mean ± SEM.

## Results

### Effects of LPS exposure at different time points postnatally on the timing of pubertal onset

**Fig. 1:**
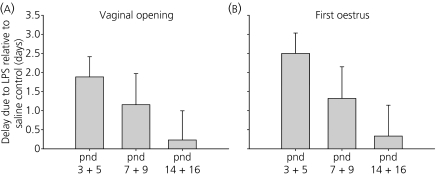
Effects of neonatal lipopolysaccharide (LPS; 50 μg/kg i.p.) at postnatal day (pnd) 3 + 5, 7 + 9 or 14 + 16 on the timing of vaginal opening (a) or first vaginal oestrus (b). Data are presented as the delay, in days, in vaginal opening or first oestrus due to neonatal-LPS treatment relative to neonatal saline treated controls. Only the neonatal-LPS treated rats on pnd 3 + 5 showed a significant delay in pubertal onset (P < 0.05). (mean ± SEM; n = 12–26 per group).

Neonatal administration of LPS on pnd 3 + 5 resulted in a significant delay in both the day of vaginal opening (Saline: 39.5 ± 0.4; LPS: 41.4 ± 0.6; P < 0.05) and day of first vaginal oestrus (Saline: 39.4 ± 0.5; LPS: 41.9 ± 0.6; P < 0.05). There were no significant differences between the LPS and saline-treated animals for the pnd 7 + 9 or pnd 14 + 16 animals (P > 0.05), despite a slight trend towards a delay in the pnd 7 + 9 group (Fig 1). None of the LPS-treated animals showed significant differences in weight compared to their respective controls (data not shown).

### Effects of neonatal LPS exposure on *Kiss1* and *Kiss1r* expression in the mPOA and ARC across puberty

**Fig. 2:**
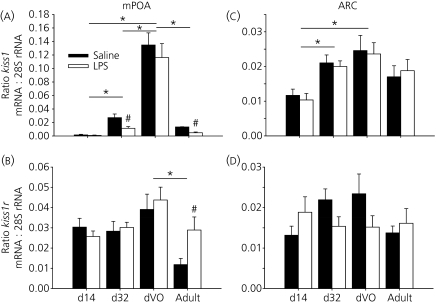
Effects of neonatal lipopolysaccharide (LPS, 50 μg/kg i.p.) or saline control at postnatal day (pnd) 3 + 5 on kisspeptin (*Kiss1*) mRNA expression in the medial preoptic area (mPOA) (a) and arcuate nucleus (ARC) (c), and on kisspeptin receptor (*Kiss1r*) mRNA expression in the mPOA (b) and ARC (d) in female rats at pnd 14, pnd 32, the day of vaginal opening (dVO) and at 11 weeks of age (Adult). *Kiss1* and *Kiss1r* mRNA levels were measured in brain micropunch samples from the mPOA or ARC using real-time reverse transcriptase-polymerase chain reaction. Quantification for *Kiss1*, *Kiss1r* and 28S rRNA was carried out on all samples; the values are expressed as a ratio of *Kiss1* mRNA and 28S rRNA, or *Kiss1r* mRNA and 28S rRNA (mean ± SEM). *P < 0.05 versus the respective treatment group at different time points; ^#^P < 0.05 versus saline control at same time point; n = 5–9 per group.

There was a significant decrease in *Kiss1* mRNA expression in the mPOA on pnd 32 (pre-puberty) and adult for animals treated with LPS neonatally, in comparison to the saline controls. Neither the pnd 14 nor dVO groups showed a significant change in *Kiss1* mRNA expression as a result of neonatal LPS administration. The levels of *Kiss1* mRNA expression differed significantly between the pnd 14, pnd 32 and dVO groups, increasing with age; in both LPS and saline-treated groups the dVO expression of *Kiss1* was significantly higher than in adults (P < 0.05; Fig.2a).

There was no significant difference in *Kiss1r* mRNA expression across the pubertal transition period, and no significant effect from LPS treatment for pnd 14, pnd 32 or dVO. There was, however, a significant decrease in *Kiss1r* mRNA expression for the adult animals in comparison to the dVO group, for both LPS and saline-treated groups, and a significant up-regulation of *Kiss1r* mRNA expression in the mPOA in adult animals in response to neonatal LPS treatment (P < 0.05; Fig. 2b).

In the arcuate nucleus, no significant differences in *Kiss1* mRNA expression were observed between the LPS and saline-treated groups at any time point. There was a significant difference in *Kiss1* mRNA expression between the pnd 14 and pnd 32 groups, and between the pnd 14 and dVO groups, with the expression increasing with age, but no significant up-regulation between pnd 32 and dVO (P > 0.05; Fig. 2c).

**Fig. 3:**
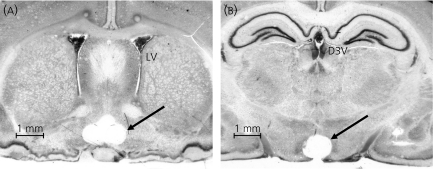
Representative examples of 300 μm coronal sections of pnd 77 (Adult) rat brain stained with crystal violet, showing the position of the punched medial preoptic area (mPOA) at approximately anteroposterior −0.28 mm from bregma (a) and arcuate nucleus at approximately −4.16 mm from bregma (b). Lateral ventricles (LV) and the dorsal third ventricle (D3V) are also indicated.

*Kiss1r* mRNA expression in the arcuate nucleus showed no significant change either with age or as a result of LPS treatment (P < 0.05; Fig. 2d). Representative examples of mPOA and ARC punched brain sections from pnd 77 (Adult) rats are shown in Fig. 3; the ARC punches shown also contain small extra-arcuate cell populations including the median eminence. None of the LPS-treated animals showed significant differences in weight compared with their respective controls (data not shown).

## Discussion

The present experiments demonstrate that there is a critical window for the programming of the developing HPG axis by neonatal immune challenge with LPS, and that such an insult has a significant effect on the timing of puberty and the expression of *Kiss1* mRNA in the mPOA during puberty. The effect on pubertal timing was only significant when LPS treatment was given on pnd 3 + 5. Of note is the absence of effect of neonatal LPS treatment on body weight (present study, 24), a factor that is well known to affect the timing of pubertal onset.

There have been few other studies of this phenomenon, although a recent study by Iwasa *et al.* (25) reported that, in rats injected with LPS at postnatal day 10, there was no difference in oestrous cycle length of adult females between neonatally LPS- and saline-treated animals, supporting our findings that an immune challenge after pnd 7 does not overtly affect reproductive function.

We have shown for the first time that neonatal treatment with LPS on postnatal days 3 + 5 results in a significant decrease in *Kiss1* mRNA expression in the mPOA on pnd 32, but no changes in Kiss1r mRNA expression. This decrease in *Kiss1* mRNA expression could provide a mechanism for the observed delay of puberty. By contrast, the lack of effect of neonatal LPS treatment on *Kiss1* or *Kiss1r* expression in the ARC would indicate that kisspeptin/Kiss1r signalling in this brain region is not an obvious contributing factor to the pubertal delay. The high levels of *Kiss1* mRNA in the combined mPOA and ARC of the pnd 32 groups compared to the pnd 14 groups corroborate findings by Navarro *et al.* (13) that kisspeptin is expressed at a basal level between pnd 10 and pnd 25 in the whole hypothalamus, and is up-regulated several days before vaginal opening. Navarro *et al.* (13) observed high expression on pnd 30, which corresponds closely to our chosen time point of pnd 32, taking into account that we observed vaginal opening on approximately pnd 38, compared to their pnd 35 for untreated animals. By contrast to the further marked rise in *Kiss1* expression between pnd 32 and the day of VO in the mPOA, the kisspeptin neurones in the ARC do not appear to undergo changes during this peripubertal period.

In the present study, *Kiss1r* mRNA expression showed no significant changes between pnd 14 and pnd 32, unlike the marked up-regulation of the receptor observed by Navarro *et al.* (13). This difference may reflect the contrast between analysing specific brain nuclei rather than the entire hypothalamus, but nevertheless remains puzzling. Herbison *et al.* (19) have shown an absence of a change in *Kiss1r* expression in preoptic area GnRH neurones during pubertal development in the male mouse. However, Plant *et al.* (26) have reported an increase in *Kiss1r* mRNA expression across the pubertal transition in the mediobasal hypothalamus in female rhesus monkeys, although they show no differences in *Kiss1r* mRNA expression between juveniles and pubescent males despite an increase in *Kiss1*.

Although we have shown very early neonatal exposure to LPS may delay puberty as a results of long-term changes in *Kiss1* mRNA expression in the mPOA, the underlying mechanism of action of LPS remains to be established. LPS acts to provoke a febrile immune response through the production of cytokines which stimulate the production of cyclooxygenase-2 and PGE2 (4). PGE2 administration in neonates has been shown to mimic the action of oestradiol in orchestrating sexually dimorphic neuronal organisation in the preoptic area, and is linked to masculinisation of sexual behaviour in female rats (27, 28). Because the LPS administration on pnd 3 + 5 falls within a critical window during which this sexual dimorphism in the preoptic has been shown to arise (29, 30), it is possible that there is a link between the action of PGE2 and an alteration of the normal sexual dimorphism of the kisspeptin neurones in the AVPV; it has previously been demonstrated that neonatal androgen administration in the rat plays a role in determining the sexual dimorphism in the AVPV, but not the ARC (31). It has yet to be determined whether PGE2 affects the sexually dimorphic organisation of the kisspeptin neurones, but if this is the case, it offers a possible explanation for the lack of long-term effects on the HPG axis as a result of LPS treatment on pnd 7 + 9 and 14 + 16 because the timing of these injections may fall outside the (currently unknown) critical period during which the differentiation takes place. Further work is required to address these issues.

In adult animals, we found a significant increase in mPOA *Kiss1r* mRNA expression in response to neonatal LPS administration. This up-regulation of kisspeptin's receptor may represent a compensatory effect to allow some reproductive function despite reduced *Kiss1* expression possibly through increased detection of the ligand by increased receptor presence, although, at the present time, this is just speculation. Nevertheless, we observed the same down-regulation of *Kiss1* and corresponding up-regulation of *Kiss1r* in response to both acute and chronic corticosterone administration in ovariectomised adults, although, in contrast to neonatal LPS treatment, corticosterone significantly alters expression profiles in both the mPOA and ARC (21). We have previously shown that adult animals treated neonatally with LPS exhibit chronic hypercorticosteronaemia, as well as persistent elevation in response to a mild stressor (6), but neither acute nor chronic stress levels of corticosterone had any effect on pulsatile LH secretion (21). The possible compensation mechanism for up-regulating *Kiss1r* in the presence of low *Kiss1* levels in adults would agree with previous reports that, under nonstressed conditions, pulsatile LH secretion is unaffected by neonatal treatment with LPS in ovariectomised females (7), suggesting that the altered expression of *Kiss1* and its receptor in the mPOA does not affect the basal operation of the HPG axis, at least in agonadal female rats. However, we have observed that pnd 3 + 5 LPS treatment has a disruptive effect on oestrus cyclicity, with many more LPS than saline-treated animals exhibiting irregular oestrus cycles, both immediately post-puberty and as adults (32), indicating that the change in *Kiss1*/*Kiss1r* expression profiles as a result of neonatal LPS may be the first manifestation of more long-lasting changes to the HPG axis, namely the sensitisation to stress-induced suppression of the GnRH pulse generator in adulthood (7).

The present study is the first to demonstrate that a neonatal immune challenge can have direct effects on the timing of puberty, concordant with altered hypothalamic *Kiss1* expression. That such an early insult is capable of such long-term effects is not entirely unexpected. Many studies of perinatal programming (33) have contributed to the understanding that adverse early environments can have long-lasting effects on adult health, with these changes corresponding to the stages of brain development that continue to mature both before and after birth. Similar to many epigenetic factors that influence development, immunological challenge has come under scrutiny recently, and neonatal LPS administration has been discovered to programme a wide range of adult phenotypes, including an attenuation of the febrile response (34), insulin sensitivity (35), susceptibility to stress-induced suppression of reproductive function (7), and adult anxiety-related behaviour (36). It is apparent that a stressful environment in the perinatal period can program an animal's immune, metabolic and reproductive functions later in life; it remains to be shown which changes are adaptations that allow the animals to better cope with a stressful adult environment, and which represent disadvantages resulting from disruption at key points in development.

## References

[1] Shanks N, Larocque S, Meaney MJ (1995). Neonatal endotoxin alters the development of the hypothalamic-pituitary-adrenal axis: early illness and later responsivity to stress. J Neurosci.

[2] Breivik T, Stephan M, Brabant GE, Straub RH, Pabst R, von Hörsten S (2002). Postnatal lipopolysaccharide-induced illness predisposes to periodontal disease in adulthood. Brain Behav Immun.

[3] Boissé L, Mouihate A, Ellis S, Pittman QJ (2004). Long-term alterations in neuroimmune responses after neonatal exposure to lipopolysaccharide. J Neurosci.

[4] Cao C, Matsumura K, Yamagata K, Watanabe Y (1995). Induction by lipopolysaccharide of cyclooxygenase-2 mRNA in rat brain; its possible role in the febrile response. Brain Res.

[5] Blatteis CM, Li S, Li Z, Feleder C, Perlik V (2005). Cytokines, PGE2 and endotoxic fever: a re-assessment. Prostaglandins Other Lipid Mediat.

[6] Shanks N, Windle RJ, Perks PA, Harbuz MS, Jessop DS, Ingram CD, Lightman SL (2000). Early life exposure to endotoxin alters hypothalamic-pituitary-adrenal function and predisposition to inflammation. Proc Natl Acad Sci USA.

[7] Li XF, Kinsey-Jones JS, Knox AM, Wu XQ, Tahsinsoy D, Brain SD, Lightman SL, O'Byrne KT (2007). Neonatal lipopolysaccharide exposure exacerbates stress-induced suppression of luteinizing hormone pulse frequency in adulthood. Endocrinology.

[8] Messager S, Chatzidaki EE, Ma D, Hendrick AG, Zahn D, Dixon J, Thresher RR, Malinge I, Lomet D, Carlton MB, Colledge WH, Caraty A, Aparicio SA (2005). Kisspeptin directly stimulates gonadotropin-releasing hormone release via G protein-coupled receptor 54. Proc Natl Acad Sci USA.

[9] de Roux N, Genin E, Carel JC, Matsuda F, Chaussain JL, Milgrom E (2003). Hypogonadotropic hypogonadism due to loss of function of the KiSS1-derived peptide receptor GPR54. Proc Natl Acad Sci USA.

[10] Seminara SB, Messager S, Chatzidaki EE, Thresher RR, Acierno JS, Shagoury JK, Bo-Abbas Y, Kuohung W, Schwinof KM, Hendrick AG, Zahn D, Dixon J, Kaiser UB, Slaugenhaupt SA, Gusella JF, O’Rahilly S, Carlton MBL, Crowley WF, Aparicio AJR, Colledge WH (2003). The GPR54 gene as a regulator of puberty. N Engl J Med.

[11] Gottsch ML, Cunningham MJ, Smith JT, Popa SM, Acohido BV, Crowley WF, Seminara S, Clifton DK, Steiner RA (2004). A role for kisspeptins in the regulation of gonadotropin secretion in the mouse. Endocrinology.

[12] Matsui H, Takatsu Y, Kumano S, Matsumoto H, Ohtaki T (2004). Peripheral administration of metastin induces marked gonadotropin release and ovulation in the rat. Biochem Biophys Res Commun.

[13] Navarro VM, Castellano JM, Fernandez-Fernandez R, Barreiro ML, Roa J, Sanchez-Criado JE, Aguilar E, Dieguez C, Pinilla L, Tena-Sempere M (2004). Developmental and hormonally regulated messenger ribonucleic acid expression of KiSS-1 and its putative receptor, GPR54, in rat hypothalamus and potent luteinizing hormone-releasing activity of KiSS-1 peptide. Endocrinology.

[14] Navarro VM, Fernandez-Fernandez R, Castellano JM, Roa J, Mayen A, Barreiro ML, Gaytan F, Aguilar E, Pinilla L, Dieguez C, Tena-Sempere M (2004). Advanced vaginal opening and precocious activation of the reproductive axis by KiSS-1 peptide, the endogenous ligand of GPR54. J Physiol.

[15] Plant TM, Ramaswamy S, DiPietro MJ (2006). Repetitive activation of hypothalamic G protein-coupled receptor 54 with intravenous pulses of kisspeptin in the juvenile monkey (*Macaca mulatta*) elicits a sustained train of gonadotropin-releasing hormone discharges. Endocrinology.

[16] Luan X, Zhou Y, Wang W, Yu H, Li P, Gan X, Wei D, Xiao J (2007). Association study of the polymorphisms in the KISS1 gene with central precocious puberty in Chinese girls. Eur J Endocrinol.

[17] Teles MG, Bianco SD, Brito VN, Trarbach EB, Kuohung W, Xu S, Seminara SB, Mendonca BB, Kaiser UB, Latronico AC (2008). A GPR54-activating mutation in a patient with central precocious puberty. N Engl J Med.

[18] Clarkson J, Boon WC, Simpson ER, Herbison AE (2009). Postnatal development of an estradiol-kisspeptin positive feedback mechanism implicated in puberty onset. Endocrinology.

[19] Han SK, Gottsch ML, Lee KJ, Popa SM, Smith JT, Jakawich SK, Clifton DK, Steiner RA, Herbison AE (2005). Activation of gonadotropin-releasing hormone neurons by kisspeptin as a neuroendocrine switch for the onset of puberty. J Neurosci.

[20] Cagampang FR, Maeda KI, Tsukamura H, Ohkura S, Ota K (1991). Involvement of ovarian steroids and endogenous opioids in the fasting-induced suppression of pulsatile LH release in ovariectomized rats. J Endocrinol.

[21] Kinsey-Jones JS, Li XF, Knox AM, Wilkinson ES, Zhu XL, Chaudhary AA, Milligan SR, Lightman SL, O'Byrne KT (2009). Down-regulation of hypothalamic kisspeptin and its receptor, Kiss1r, mRNA expression is associated with stress-induced suppression of luteinising hormone secretion in the female rat. J Neuroendocrinol.

[22] Paxinos G, Watson C (1986). The Rat Brain in Stereotaxic Coordinates.

[23] Palkovits M (1973). Isolated removal of hypothalamic or other brain nuclei of the rat. Brain Res.

[24] Spencer SJ, Mouihate A, Galic MA, Ellis SL, Pittman QJ (2007). Neonatal immune challenge does not affect body weight regulation in rats. Am J Physiol Regul Integr Comp Physiol.

[25] Iwasa T, Matsuzaki T, Murakami M, Kinouchi R, Shimizu F, Kuwahara A, Yasui T, Irahara M (2009). Neonatal immune challenge affects the regulation of estrus cyclicity and feeding behaviour in female rats. Int J Devl Neuroscience.

[26] Shahab M, Mastronardi C, Seminara SB, Crowley WF, Ojeda SR, Plant TM (2005). Increased hypothalamic GPR54 signaling: a potential mechanism for initiation of puberty in primates. Proc Natl Acad Sci USA.

[27] Amateau SK, McCarthy MM (2002). A novel mechanism of dendritic spine plasticity involving estradiol induction of prostaglandin-E2. J Neurosci.

[28] Amateau SK, McCarthy MM (2004). Induction of PGE2 by estradiol mediates developmental masculinization of sex behavior. Nat Neurosci.

[29] Davis EC, Popper P, Gorski RA (1996). The role of apoptosis in sexual differentiation of the rat sexually dimorphic nucleus of the preoptic area. Brain Res.

[30] Davis EC, Shryne JE, Gorski RA (1996). Structural sexual dimorphisms in the anteroventral periventricular nucleus of the rat hypothalamus are sensitive to gonadal steroids perinatally, but develop peripubertally. Neuroendocrinology.

[31] Kauffman AS, Gottsch ML, Roa J, Byquist AC, Crown A, Clifton DK, Hoffman GE, Steiner RA, Tena-Sempere M (2007). Sexual differentiation of Kiss1 gene expression in the brain of the rat. Endocrinology.

[32] Wu XQ, Li XF, Popat N, Milligan SR, O'Byrne KT (2008). Neonatal Exposure to Lipopolysaccharide Disrupts Ovarian Function in the Adult Rats.

[33] Seckl JR, Meaney MJ (2004). Glucocorticoid programming. Ann NY Acad Sci.

[34] Spencer SJ, Martin S, Mouihate A, Pittman QJ (2006). Early life immune challenge: defining a critical window for effects on adult responses to immune challenge. Neuropsychopharmacology.

[35] Nilsson C, Jennische E, Ho HP, Eriksson E, Bjorntorp P, Holmang A (2002). Postnatal endotoxin exposure results in increased insulin sensitivity and altered activity of neuroendocrine axes in adult female rats. Eur J Endocrinol.

[36] Spencer SJ, Heida JG, Pittman QJ (2005). Early life immune challenge--effects on behavioural indices of adult rat fear and anxiety. Behav Brain Res.

